# Supplementary figures and images for: Zebrafish do not have a calprotectin ortholog
Source: PLoS One. 2025 May 2;20(5):e0322649. doi: 10.1371/journal.pone.0322649 (PMC12047837; doi:10.1371/journal.pone.0322649)

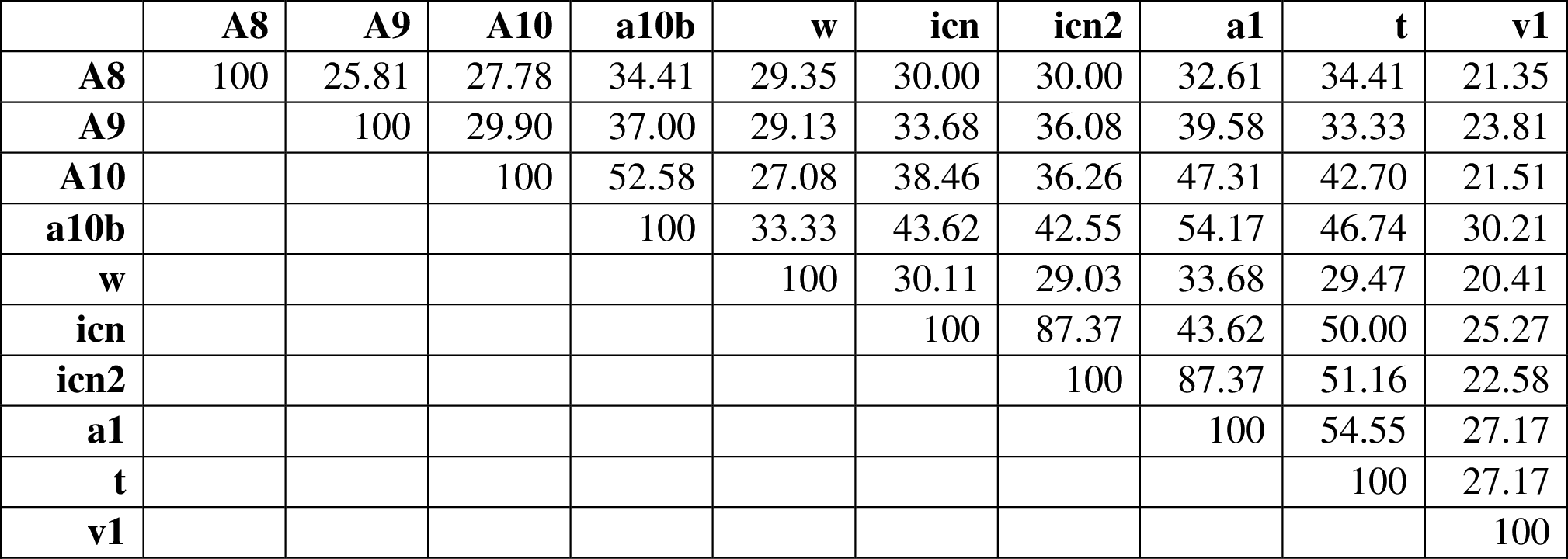

Supplement: S1 Table — Human S100 proteins (A8, A9, and A10) and homologs from zebrafish (a10b, w, icn, icn2, a1, t, and v1). (TIF) [file pone.0322649.s001.tif]

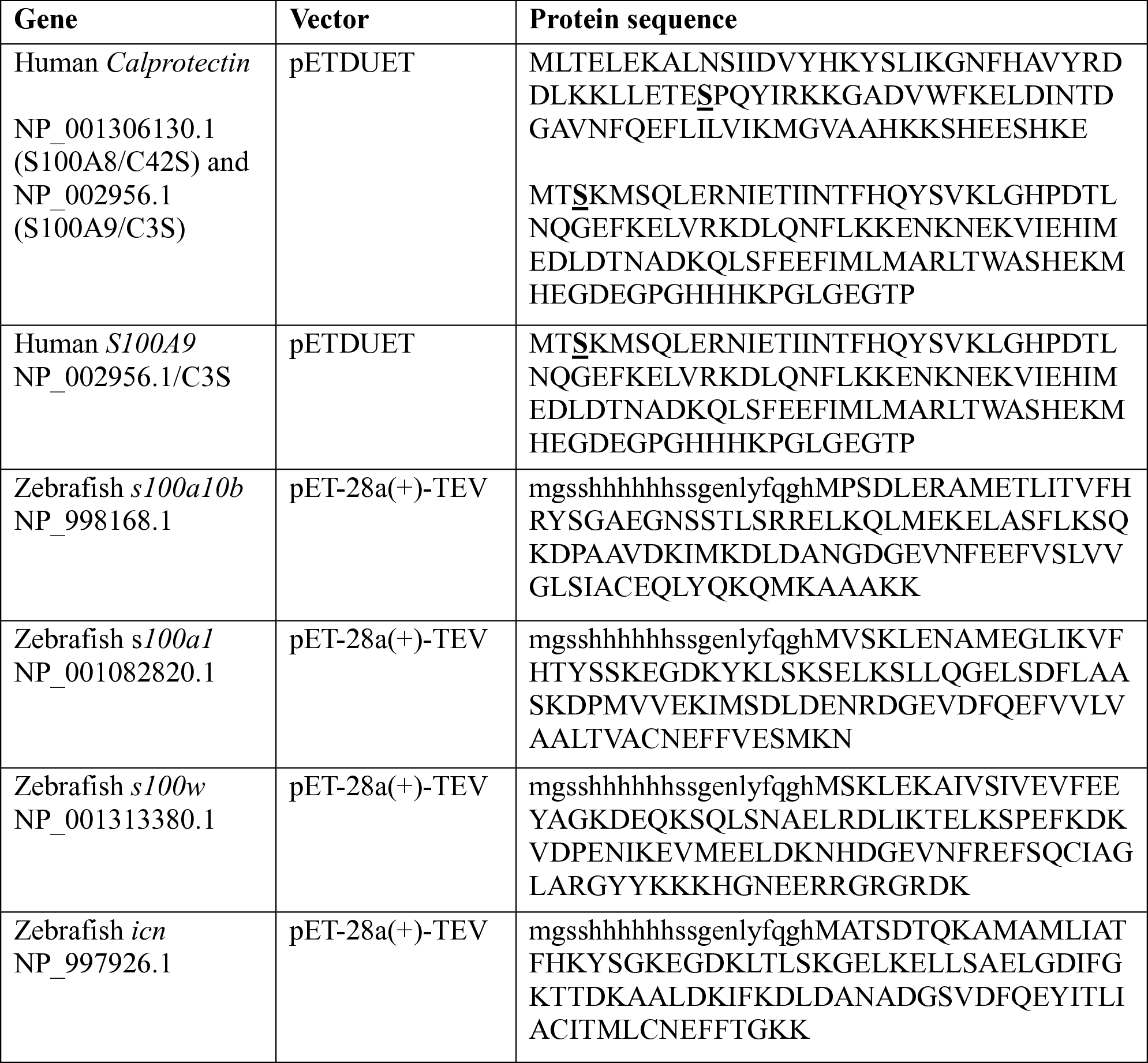

Supplement: S2 Table — Lowercase amino acids indicate the addition of a 6xHis tag and TEV protease cleavage site. Bold and underlined amino acids are mutations relative to the reference sequence. (TIF) [file pone.0322649.s002.tif]

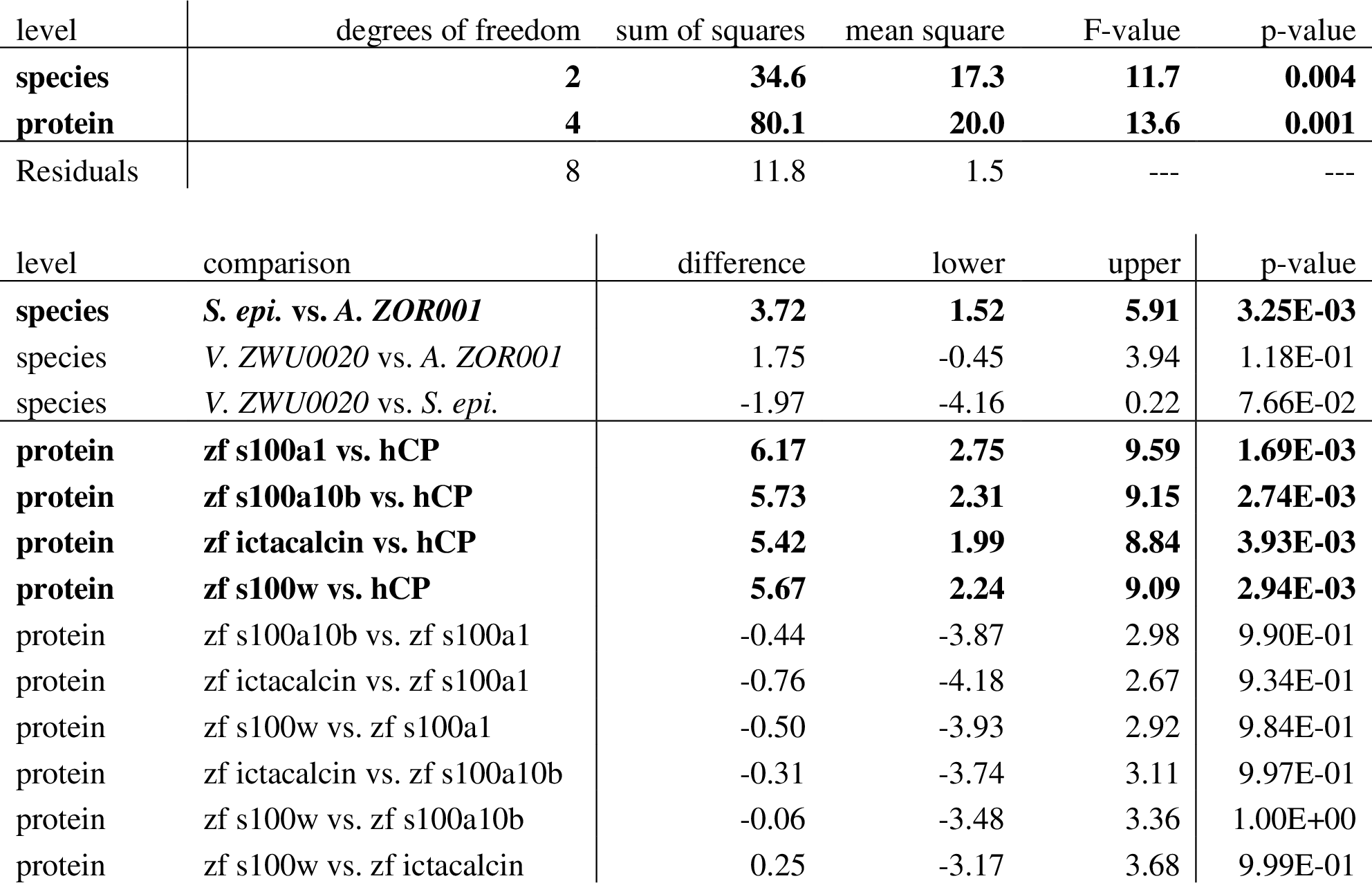

Supplement: S3 Table — The top sub-table shows the correlation of bacterial species (S. epi., A. ZOR001, and V. ZWU0020) and protein (hCP, s100a1, s100a10b, ictacalcin, s100w) with the mean change in area under the growth curves for the 50 μM treatment condition. The bottom sub-table shows the results of a post hoc Tukey test applied to the ANOVA results. This test reveals that the effect of hCP is significantly different than any of the zebrafish proteins (p values between 0.00169 and 0.0039; bolded rows). The effects of the zebrafish proteins cannot be distinguished. (TIF) [file pone.0322649.s003.tif]

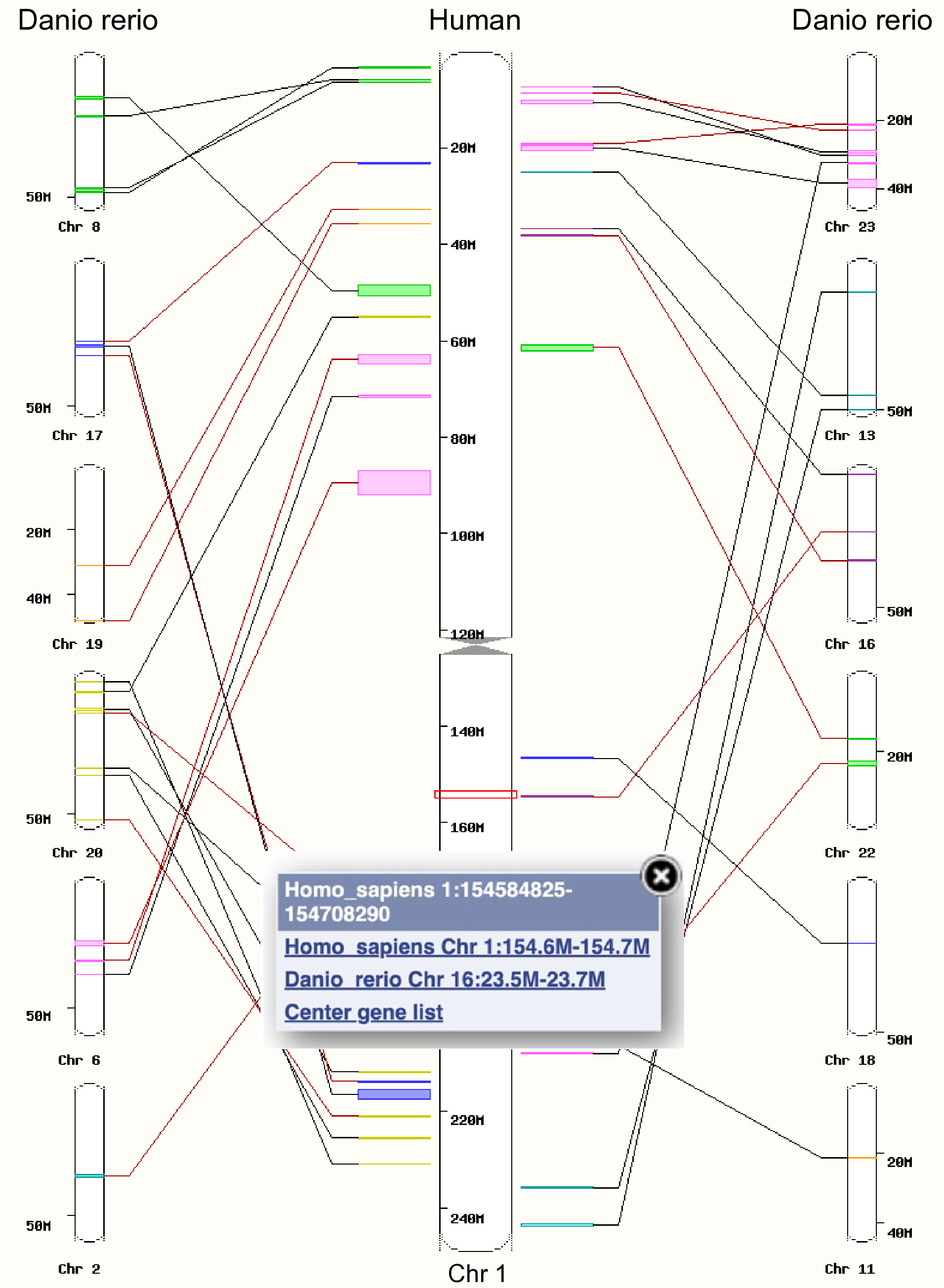

Supplement: S1 Fig — Central chromosome is human chromosome 1; outer chromosomes are zebrafish chromosomes with regions syntenic to regions of human Chr1. The red box on Chr1 indicates the region containing 19 of the 24 human S100 genes (1:154584825–154708290). This is syntenic to zebrafish Chr16. (TIF) [file pone.0322649.s004.tif]

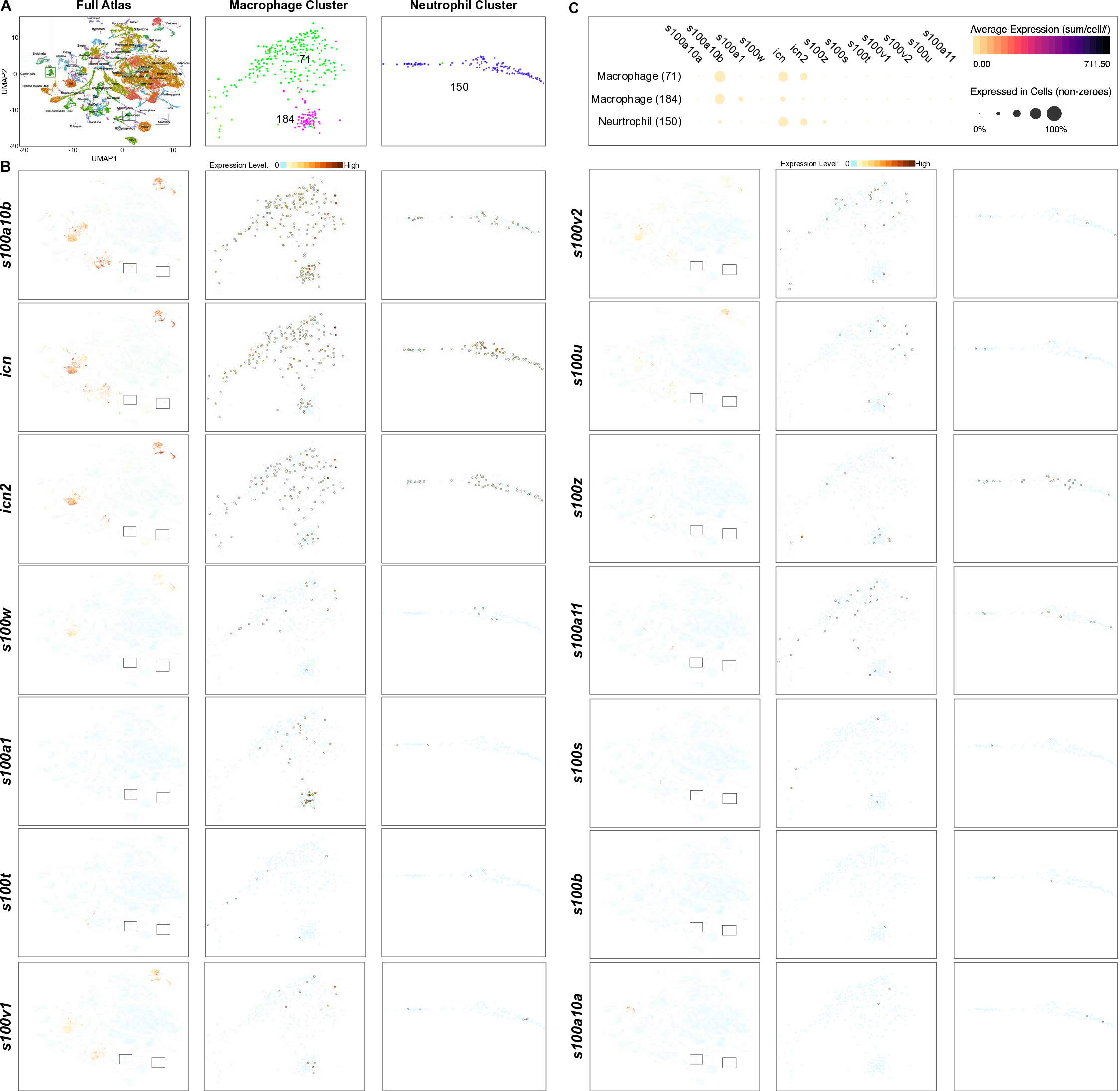

Supplement: S2 Fig — Developmental scRNAseq datasets taken from the UCSC cell browser NCBI Bioproject: 564810. A) This set of 3 atlases shows the layout that will be used for panel B. Each point is a cell from 1-, 2-, or 5-days post-fertilization zebrafish, separated by transcriptional profile along the UMAP1 and UMAP2 axes. The “Full Atlas” on the far left shows how all the cells in the dataset are related to one another; the middle- and right-most atlases zoom in on the regions corresponding to macrophage clusters 71 and 184 and neutrophil cluster 150. These regions are shown by the small boxes on the full atlas. B) Each row of atlases corresponds to a different zebrafish s100 RNA (labeled on the left). Cells are colored by the relative expression level of that RNA shown by the legend at the top (blue: no expression; red: high expression). C) A dot plot summarizes which zebrafish s100s are expressed in the macrophage and neutrophil clusters. Darkness of dot color represents average expression. Dot size indicates the percent of cells within the cluster that express the RNA. The authors performed differential gene expression analysis using the FindAllMarkers function in Seurat v3.4.4 using Wilcoxon rank sum test. (TIF) [file pone.0322649.s005.tif]

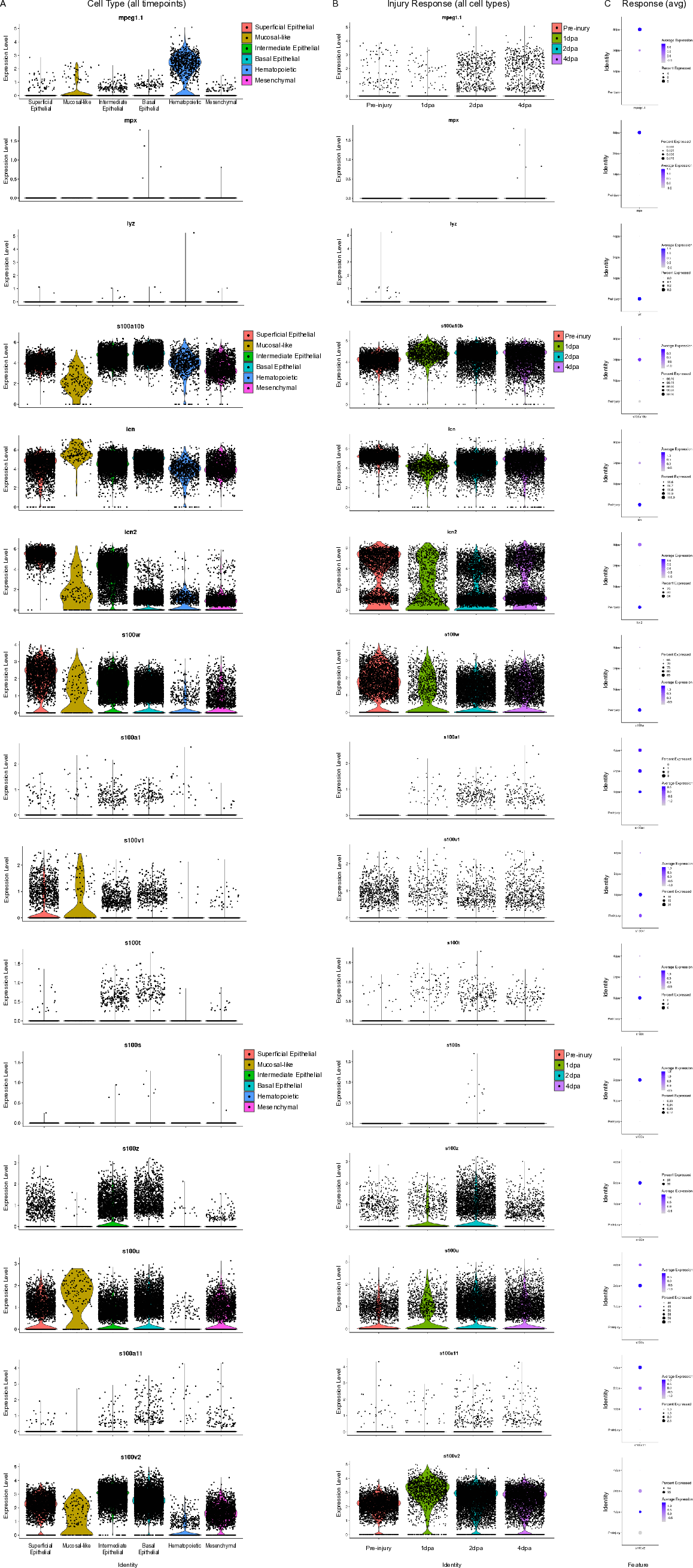

Supplement: S3 Fig — Data are taken from NCBI GEO accession number GSE137971 [44]. We accessed the data published at https://k326xh.shinyapps.io/FinRegenerationSCRNA/ on June 11th, 2024. The authors state that relative RNA expression level was found using Seurat v3.0 by comparing the expression profiles of the specified gene with those of the rest of the cells using Wilcoxon rank sum–based approach with the criteria of log fold change more than 0.25 and a minimum cell percentage of 0.25. A) The relative expression level of specific RNAs (y-axis) within certain cell types (x-axis; also denoted by color in legend) across all stages, pre-injury through 4dpa. Individual cells are shown as points. B) The relative expression of each s100 RNA (y-axis) within a specific stage of regeneration, e.g., pre-injury, 1-day post-amputation (dpa), 2dpa, or 4dpa (x-axis; also denoted by color in legend) across all cell types. C) Dot plots summarize the data in B. Dot size represents the percent of cells within the cluster expressing the gene. Dot color indicates the average expression level of the particular RNA within the cluster compared to its expression level in cells in the rest of the dataset using a Wilcoxon rank sum–based approach. (TIF) [file pone.0322649.s006.tif]

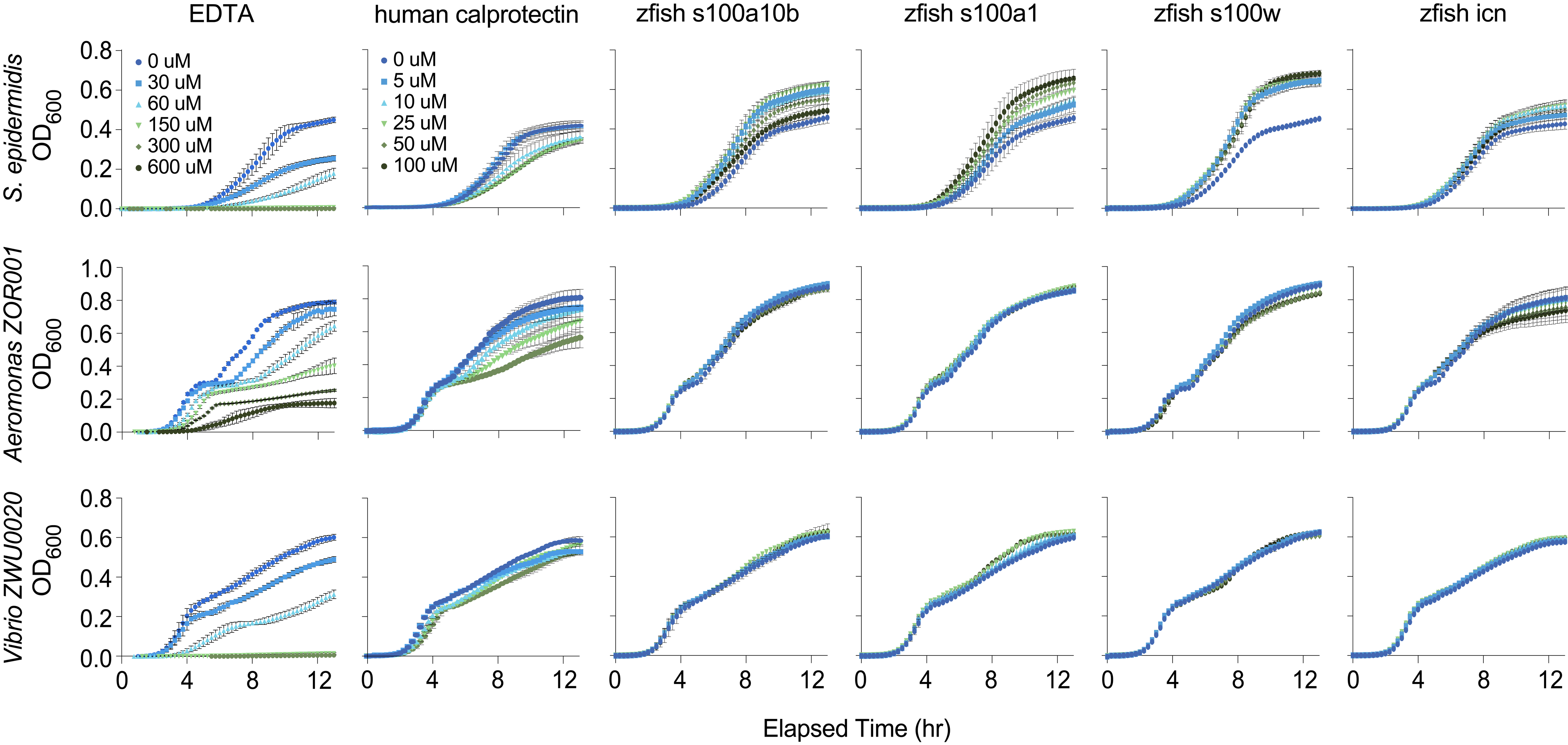

Supplement: S4 Fig — Rows show assays done with the bacterial strain indicated on the left. Columns show results for the S100 protein indicated at the top. Bacterial growth was measured over 13 hours in the presence of S100 concentrations ranging from 0–100 μM, dark blue to dark green as shown in the legend at the top left. A control experiment was done using EDTA at concentrations equal to the ratio of the number of metal binding sites per calprotectin heterodimer. 6 binding sites total per dimer: 4 calcium-binding sites and 2 zinc-binding sites. All measurements were done in biological triplicate of technical triplicates except s100A1 at 100 μM which only contains data from two biological replicates. Datapoints and error bars represent the mean and standard error of biological replicates. (TIF) [file pone.0322649.s007.tif]

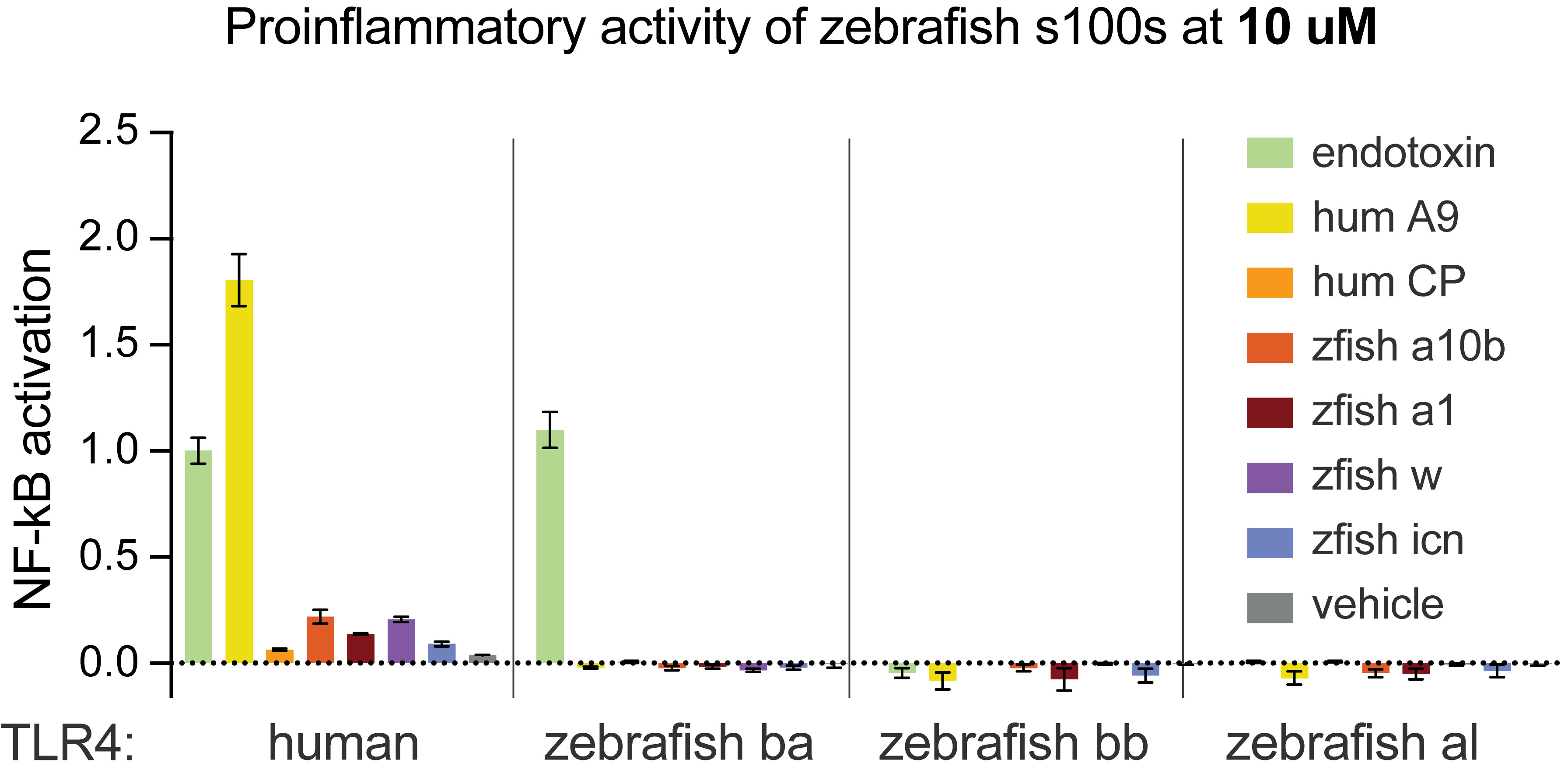

Supplement: S5 Fig — Bars show the average signal across three biological replicates, with error bars indicating standard error. The positive controls for this experiment included human TLR4 and zebrafish Tlr4ba treated with endotoxin (green), and human TLR4 treated with 1 μM human S100A9 (yellow). For zebrafish experiments, we used 10 μM protein. There is no known agonist for zebrafish Tlr4bb and Tlr4al complexes. All data was background subtracted and normalized to the signal from human TLR4 treated with endotoxin. (TIF) [file pone.0322649.s008.tif]

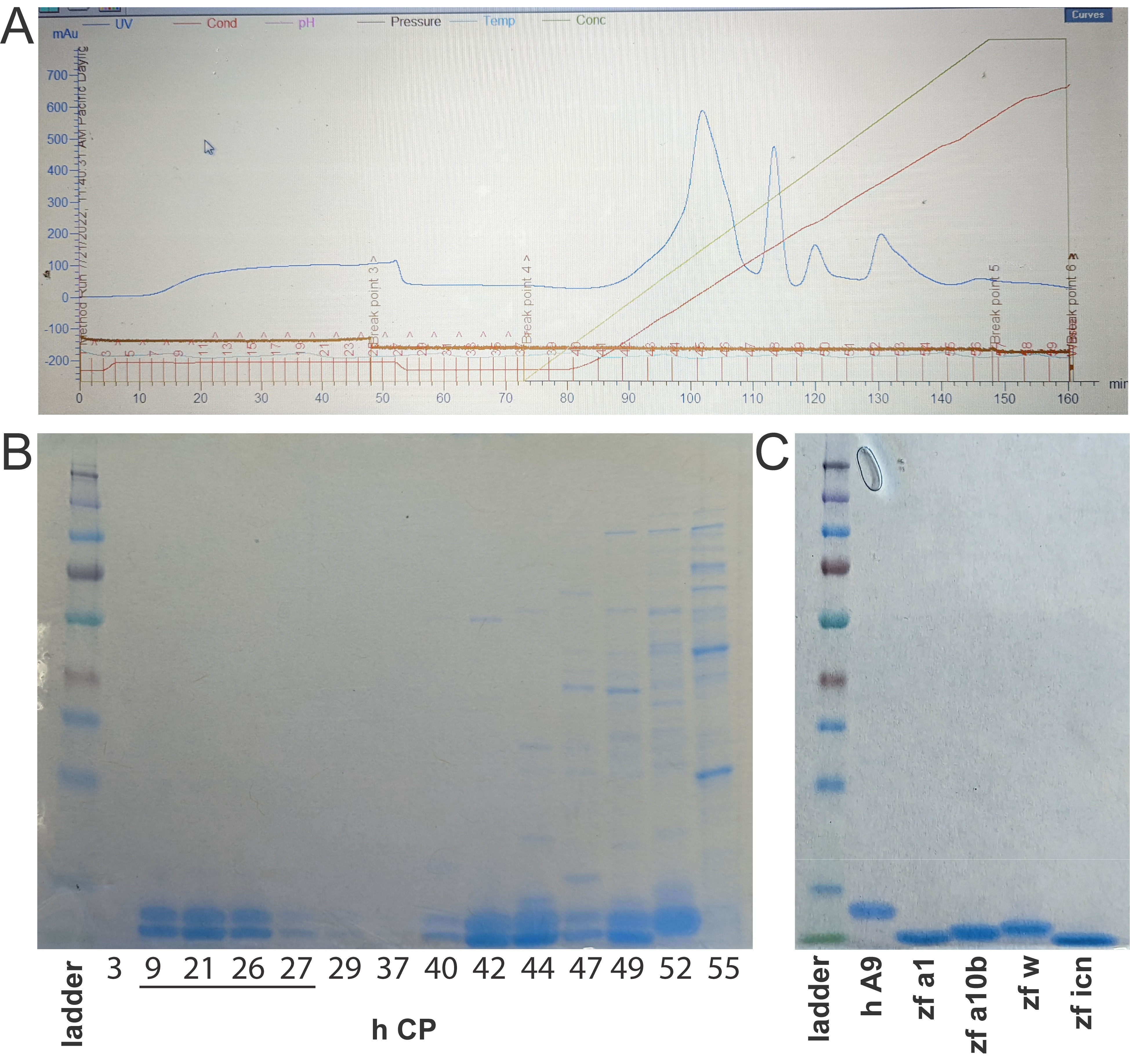

Supplement: S6 Fig — A) The chromatogram of the final anion exchange chromatography step for the purification of human calprotectin. Calprotectin flowed through the column during the low salt wash step while S100A9 and other proteins stuck to the column and were eluted later in high salt buffer. B) An SDS-PAGE analysis of fractions corresponding to the chromatogram (red numbers). Fractions 8–27 (underlined) show a ~ 1:1 ratio of S100A8 (bottom band) to S100A9 (top band) and were pooled for use as pure calprotectin. C) A gel showing purified human S100A9 and zebrafish s100a1, s100a10b, s100w, and icn. The ladder in both gels is the Spectra™ Multicolor Broad Range Protein Ladder. S100 proteins fall between ~10 kDa (green ladder band at bottom) and ~ 15 kDa (lowest blue ladder band). (TIF) [file pone.0322649.s009.tif]

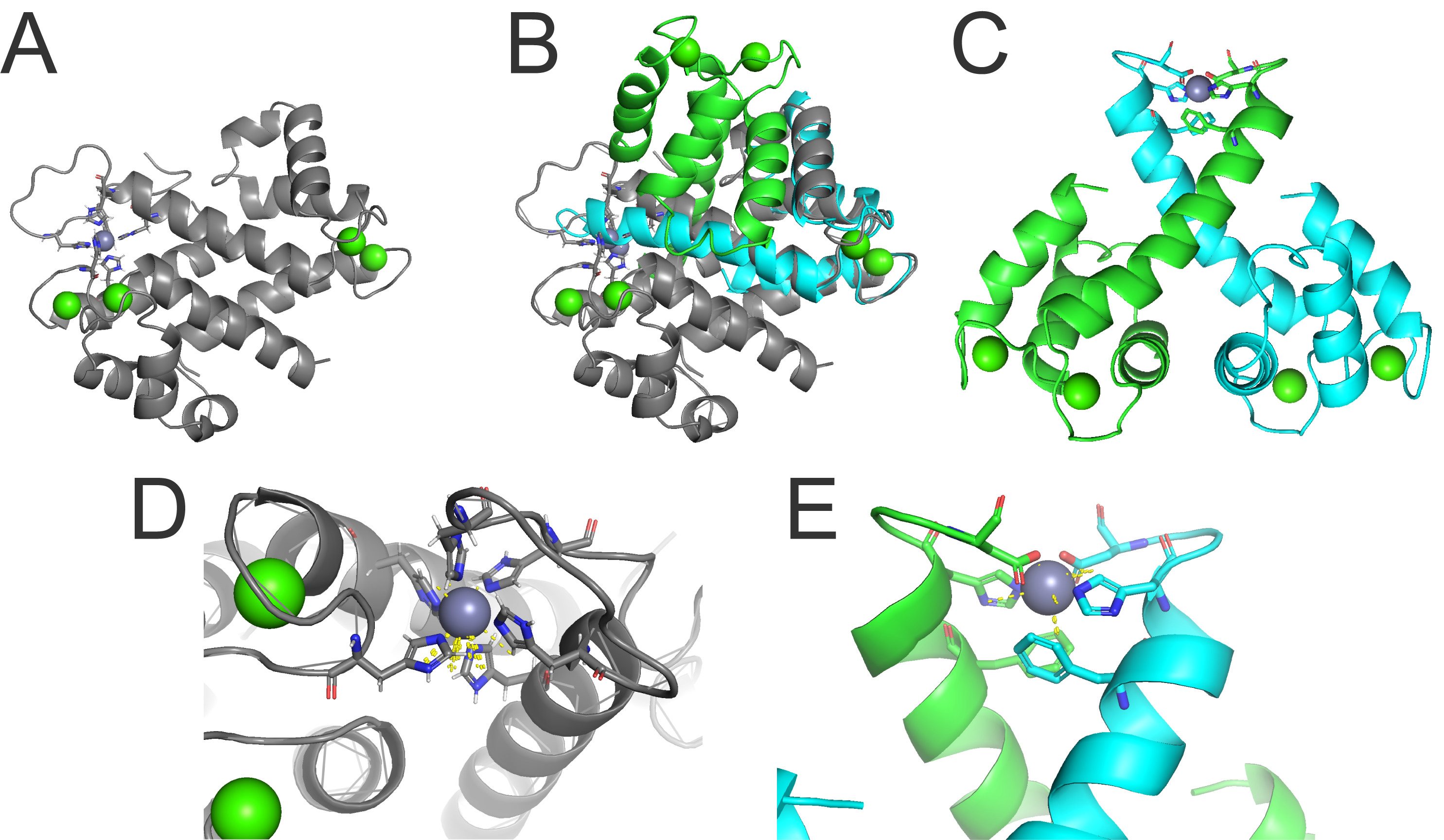

Supplement: S7 Fig — A) The calprotectin heterodimer (gray) from crystal structure PDB: 8JSC. Calcium ions are represented as green spheres and the zinc ion as a purple sphere. B) Zebrafish s100t homodimer aligned to calprotectin heterodimer. C) Zebrafish s100t homodimer. D) A close-up of calprotectin’s hexahistidine site coordinating a zinc ion via polar contacts (dashed yellow lines). E) A close-up of the zebrafish s100t homodimer’s potential alternate metal binding site. Sidechains predicted to form the interaction with a zinc ion are shown as sticks with polar contacts shown. (TIF) [file pone.0322649.s010.tif]
