# Supplementary material for: Zebrafish do not have a calprotectin ortholog
Source: PLoS One. 2025 May 2;20(5):e0322649. doi: 10.1371/journal.pone.0322649 (PMC12047837; doi:10.1371/journal.pone.0322649)
Supplement: Attachment — Submitted filename: Response to Reviewers.docx [file pone.0322649.s015.docx]

**Reviewer 1:** Comment: The authors make a definitive statement in the title and in the text that zebrafish do not express calprotectin, without providing more extensive evaluation, even as they place the onus on other investigators in the field to prove that such a function exists. They evaluate the functions of only four of the nearly 14 or more S100 genes in zebrafish, some of which could be teleost-specific without ortholog equivalents in mammals. Without further extensive studies it cannot therefore be concluded that they mediate or do not mediate innate immune responses akin to calprotectin. At minimum, the title and discussion must be modified to be more reflective of the limitations of the current study as suggested below. (--suggestions moved later in responses: noted as Comment 1A and 1B)

Thank you for pointing this out. We have updated the title, several lines of text, and added a section (line 212, subheading: “Could a calprotectin paralog have convergently evolved similar immune functions?”). These changes reflect that we know for certain that there is no ortholog of calprotectin in zebrafish, but we cannot rule out that a s100 or other protein may have convergently evolved functions akin to calprotectin. We have emphasized the nuance between proving a protein is the same protein in different species, versus establishing convergent functional properties between different proteins. We hope we have now made this important distinction clear and that we drive home the point that zebrafish did not inherit an ortholog of the calprotectin protein and therefore do not have calprotectin.

We have also modified the text to highlight that the experiments in this manuscript test only the possibility of functionally convergent evolution in a fraction of the potential zebrafish s100 protein complexes. There is much work to be done on this front; the volume of work to rigorously establish the various roles of zebrafish s100 homo- and hetero-dimers and their implications in health and disease, let alone their antibacterial and proinflammatory abilities, could fill a series of manuscripts. We have now elaborated on this in the text in a new section (line 418, subheading “Structure predictions do not support convergent evolution of a calprotectin-like metal chelation site in zebrafish s100s”) as well as in the discussion (line 518).

**Reviewer 2:** Comment 1) As the authors rightly pointed out in the discussion, calprotectin is an heterodimer and it has been unambiguously shown in mammals that the A8 and A9 homodimers have distinct properties than the heterocomplex, both for inflammatory and antimicrobial properties. I therefore believe that a proper comparison cannot bypass this point and heterodimerization of zebrafish S100 proteins, at least for the ones present in immune cells and investigated in this study, must be evaluated. This can be easily monitored using the split GFP trap method as described in Spratt et al FEBS J 2018 (doi:10.1111/febs.14775). If any of these proteins are able to form heterodimers, then the antimicrobial and NFκB-activation assays should be repeated with the heterocomplexes.

We have considered experiments to explore zebrafish heterodimer s100 activities. There are two reasons for which we chose not to pursue this:

1) Due to the lack of phylogenetic evidence that any zebrafish s100 is orthologous to S100A8 or S100A9, we focused on testing the functional relevance of previous claims that zebrafish s100a10b is calprotectin—s100a10b cannot be calprotectin due to its phylogenetic history but could have convergently evolved metal chelating and/or immune stimulatory abilities that could be relevant to disease model research in zebrafish.

2) As this reviewer pointed out in comment #9, in order to form a functional calprotectin complex, both S100A8 and S100A9 must be expressed from the same plasmid in E. coli; you cannot simply mix two preparations of homodimers. This means that even just testing all combinations of the four promising zebrafish s100s candidates we identified would require expressing, purifying, and functionally testing six additional protein complexes.

As noted by both reviewers, there are at least 14 s100s in zebrafish. Due to the complicated differences in the evolutionary history of mammalian and teleost s100s, it is not obvious which heterocomplex would be the most likely to have convergently evolved bactericidal and/or proinflammatory properties. In our previous response to reviewer 1 we noted that a large a body of work, which is out of the scope for this manuscript, would be to experimentally explore the functions of all 196 zebrafish s100 homo- and hetero-complexes.

We have now included a section describing a rough structural evaluation of potential zebrafish s100 dimer complexes using AlphaFold3 and PyMol (see line 418, subheading: “Structure predictions do not support convergent evolution of a calprotectin-like metal chelation site in zebrafish s100s”).

Our first pass efforts to reveal convergent evolution within the zebrafish s100 complexes was not fruitful but does not diminish the fact that there is no ortholog of calprotectin in zebrafish (i.e. zebrafish do not have “calprotectin”). We have attempted to drive this point home more clearly throughout the text.

**Reviewer 2:** Comment 5) The antimicrobial activity of calprotectin is linked to its unique hexahistidine site that chelates metal ions with high affinity. The authors chose to focus on zebrafish S100 proteins that are expressed in immune cells. But are there any zebrafish S100 proteins that may resemble more calprotectin for this particular function, and notably possess a high content of histidine residues in their C-terminal region? This should be investigated at least computationally and discussed in the manuscript.

Thank you for this interesting suggestion. The crystal structure of human calprotectin bound to zinc (PDB: 8SJC) illustrates the residues that make up the hexa-histidine site that this reviewer refers to. The residues that constitute the hexa-histidine site are two residues from human S100A8: H17 and H27 within the N-terminal helix and loop region, as well as four residues from human S100A9: H87, H91, H99 and H101 at the C-terminus.

In an alignment of all teleost s100s from Kraemer et al., 2008 (Additional File 4), s100b (including Danio rerio s100b) has 3 histidine residues at its C-terminus which is the highest concentration out of all teleost s100 proteins. Zebrafish s100b also has two N-terminal histidine residues at positions 16 and 39. We have investigated this idea more thoroughly using the AlphaFold3 structure prediction software. We have included an evaluation of all predicted structures of homo- and hetero-dimers and highlighted potential sites for metal chelation in the new results section, “Structure predictions do not support convergent evolution of a calprotectin-like metal chelation site in zebrafish s100s” (line 418), included a new figure, “Fig 5. Predicted structures of zebrafish s100 homodimers and heterodimers” (line 434), and added supplementary figure S7.

**Reviewer 1:** Comment 1A. Physical injury models of acute inflammation in zebrafish have limitations, and therefore other models of inflammation including chemically induced colon inflammation models, and pathogenic bacterial and viral infection models are being developed and validated in zebrafish which could provide more in-depth analysis of the role of zebrafish s100 proteins in promoting innate immune responses.

An insightful suggestion. We have now included references to reviews on zebrafish inflammation models [line 526].

**Reviewer 2:** Comment 2) For the antimicrobial assay, the authors chose three representative pathogens. While Vibrio species have already been investigated in zebrafish for their link with S100 proteins, the two others haven’t. It is in my opinion important to also test E. tarda for which a clear modulation of S100 genes in response to infection has already been observed.

It would be interesting to explore whether zebrafish s100s previously noted to be transcriptionally upregulated during the immune response to infection by the serious fish pathogen, *Edwardsiella tarda*, have any ability to directly affect *E. tarda* growth. For example, Zhang et al., 2019 noted that transcripts of s100a1, s100a10b, icn1, icn2, s100b, and s100v1 were significantly upregulated in the gut and spleen 3-6 hours post-infection with *E. tarda*. To our knowledge there has not been a direct study on *E. tarda*’s response to calprotectin as it is not a major human pathogen and zebrafish do not have calprotectin, therefore it was not considered a standard to be tested against.

Although this could be useful knowledge for the field of fish pathogen research, we do not believe this particular experiment is necessary to prove that the four studied zebrafish s100 proteins lack the broad-spectrum antimicrobial activity of metal ion chelation which calprotectin employs. In our antimicrobial assay, we show that human calprotectin can inhibit growth, in a dose-dependent manner, of three bacterial strains. We used these strains in part because they were accessible at our institution. The human-derived *S. epidermidis* was chosen because it is a standard for testing calprotectin functional variants across many studies, and the two zebrafish-derived bacterial strains are related to human pathogens and elicit immune responses in zebrafish. We’ve updated the text to clarify this in line 312.

**Reviewer 2:** Comment 3) I find the representation of the data for the antimicrobial assays not very intuitive, especially the choice of measuring the area under the growth curve and displaying the ΔAUC value as a measure of growth inhibition by S100 proteins is a bit odd. Why not just choose to represent the 600OD after a given incubation period against increasing concentrations of S100? This might be easier to interpret.

We wanted the reader to be able to directly compare differential effects of each protein on overall bacterial growth rather than at a snapshot in time. The Vibrio panel of Figure 3A illustrates that the endpoint OD_600_ reading is not always an accurate representation of the inhibitory effect on all phases of bacterial growth.

We thought about alternatively graphing the average growth rate for each strain challenged with increasing concentrations of all proteins, which might be easier to interpret, but would add significant noise to the data.

We also considered simply showing the growth curves of every strain/protein combination, as shown in Supplement Figure 4. However, this felt like it would be an overwhelming figure.

We have now included a more detailed explanation of the ΔAUC values and how to interpret them starting at line 334 in the results section “Zebrafish s100s do not exhibit nutritional immunity characteristics like human calprotectin”.

**Reviewer 2:** Comment 4) The antimicrobial function of mammalian S100 proteins, including calprotectin, is primarily believed to occur through metal sequestration. In the assay, the maximal concentration of S100 used was 100 µM. But the content in divalent cations of the media, as measured by ICP-MS and reported in the Methods section, is much higher than that. Zinc and iron, two central nutrients for pathogens, are for example present at 5.9 and 1.1 mM concentrations, respectively. This means that the S100 metal binding sites would be saturated, at least partly. While a weak antimicrobial activity is detected for calprotectin, such concentrations in divalent cations may hinder the true bactericidal activity of zebrafish S100 proteins if they have lower affinity for metal ions than calprotectin. The authors should provide some controls here:

**Reviewer 2:** 4a) First, a chelator of divalent cations like EDTA/EGTA should be used at the same concentration as the S100 proteins for positive control of bacterial growth inhibition.

This is a good experimental control. We initially used calprotectin as our positive control but have now included the suggested experiment in our supplemental data (S4 Fig) for completeness. This experiment was done with EDTA concentrations that match the concentration of metal binding sites in each calprotectin heterodimer challenge. The calprotectin heterodimer has 4 calcium-binding sites and 2 transition metal binding sites, so 6 uM EDTA to every 1 uM calprotectin. This control suggests that the calprotectin metal-binding sites were not saturated in our growth assays, likely due to non-saturating levels of calcium which increase calprotectin’s metal binding affinity. Nonetheless, our conclusion is the same that each strain is inhibited in growth by calprotectin under these conditions, but not by any of the zebrafish s100s tested.

**Reviewer 2:** 4b) Second, the assay should be reproduced with optimized growth media or with higher concentrations of S100 proteins to detect any potentially weak antimicrobial activity.

These are good suggestions; however, we do not believe they are necessary. Our data shows that under the conditions in our assay, human calprotectin can inhibit growth of each bacterial strain in a dose-dependent manner.

Increasing the zebrafish s100 dose to 100 uM dimer greatly exceeds the most extreme physiological extracellular calprotectin level of ~40 uM (1 mg/mL) reported in humans (Johne et al., 1997). Farr et al., 2022 reported zebrafish “calprotectin” levels up to ~2.4 uM (60 ng/mL) after infection with *Vibrio cholerae*. Our results show that even 100 uM concentrations did not exhibit antimicrobial effects for any zebrafish s100. Most of the deltaAUC curves for these proteins plateau before reaching 100 uM, suggesting that additional protein would not change our conclusion.

As our new AlphaFold3 structure predictions confirm, it is unlikely that the zebrafish proteins have a strong metal chelating site which would be necessary to support the convergent evolution of calprotectin’s nutritional immunity function.

**Reviewer 2:** 6) In the cell-based assay for NFκB activation, the authors use human S100A9 as the positive control representing mammalian S100s. Since the entire study is based on calprotectin, I find it odd that calprotectin is not employed in this assay as well. The experiment should be repeated with calprotectin for proper comparison.

This is a valid concern. In the HEK-293T cell-based assay, S100A9 is a potent activator of TLR4, and pure calprotectin is not. For this reason, we compared the TLR4 agonist ability of zebrafish s100 proteins against the well-characterized S100A9 agonist. We have now included a comparison to human calprotectin for completeness in (Figure 4, yellow bars).

**Reviewer 1:** Comment 1B. It has recently been shown that zebrafish express a low-sensitivity Tlr4/Md-2 complex that confers immune response to LPS, (although there is no ortholog in zebrafish of CD14, a co-receptor required for TLR4 signaling). Based on this report, and since calprotectin is known to bind to TLR4, the authors evaluate NF kappa B signaling in response to purified human S100A9 or purified zebrafish s100 proteins on cells expressing human TLR4 or three zebrafish tlr4 ohnologs, and show that the zebrafish s100 proteins do not elicit tlr4 mediated intracellular signaling. However, there are other cell surface receptors such as RAGE, CD36, and GPCRs that bind to S100A8/S100A9 and promote intracellular signaling. There is some evidence that orthologs of these receptors, showing conserved synteny, do exist in zebrafish.

+

**Reviewer 2:** Comment 7) TLR4 is not the sole receptor for S100A8/A9 in mammals. Obviously, RAGE is not present in zebrafish. But what about the other receptors, especially those that may preferentially bind the S100A8 moiety of calprotectin? Their presence or not in zebrafish should be brought to discussion.

This is an excellent point made by both reviewers. We have attempted to get a human RAGE reporter system working in our lab and were not successful. We have now included discussions on the hypothesis that alternate zebrafish immune receptors may be involved in the inflammatory response to s100 proteins (lines 367, 415, 493).

**Reviewer 2:** 8) No statistical analyses are provided in the manuscript. This is an important flaw of the study, in particular for the antimicrobial assays. It is important to confirm that the decrease in 600OD observed in the presence of CP for the 3 tested pathogens is statistically significant. Such statistical analyses should therefore be provided with clear description of the statistical tests employed.

Although we had stated which tests were done in the results sections (see below), we were indeed missing a clear description of our statistical analyses and significance notations in our graphs. Thank you for pointing this out.

- For the antimicrobial assays, see Supplemental Table 3 and line 333 (initial submission): “We performed an ANOVA analysis on the antimicrobial activity at 50 uM protein for every condition and found that human calprotectin had a statistically different effect than any of the zebrafish s100 proteins (p < 0.0039), but that the zebrafish proteins could not be distinguished from one another (S3 Table).”
- For the proinflammatory assays, see line 362 (initial submission): “We detected a small amount of signal for treatment with zebrafish s100a10b (orange) against human TLR4 (Bonferroni-corrected, paired two-sample t test of protein versus buffer, p value: 0.02); however, this was barely above background and much lower than the response for either endotoxin or human S100A9.”

We have now included a clear description of our statistical analyses in the methods section (line 683 and 731), updated our statistical analysis for the proinflammatory assay and added statistical significance notations in the proinflammatory figure (Fig 4, line 399).

**Reviewer 2:** 9) Many papers describe the purification of calprotectin from co-expression system as a very straightforward procedure. However, they rarely demonstrate that they obtain an heterodimer preparation devoid of contamination by A8 or A9 homodimers. The chromatogram for the purification of calprotectin on anion exchange chromatography should be displayed in Supplementary material, together with the SDS-PAGE analysis of the fractions corresponding to the A9 homodimer and the proposed A8/A9 heterodimer, for assessment of the purity and homogeneity of the protein samples.

+

**Reviewer 1:** Minor comments: a. How were the purities of the expressed zebrafish s100 proteins assessed?

Protein purity was determined to be >95% by comparing band intensities in SDS-PAGE (see line 472 of initial submission, line 562 for revised submission). We have now included example SDS-PAGE gel images including all purified proteins as well as a chromatogram and SDS-PAGE analysis for the anion exchange chromatography step for one of our calprotectin purifications (S6 Fig).

**Reviewer 2:** Minor comment: 10) Clear description of what is the ‘vehicle’ in Figure 4 should be mentioned either in the legend of the figure or in the Method section describing the NFκB activation assay.

Thank you for pointing this out. The vehicle was 200 ng/uL Polymyxin B in PBS. We have updated the figure legend (404) and methods section (line 712).
