# Supplementary material for: Zebrafish do not have a calprotectin ortholog
Source: PLoS One. 2025 May 2;20(5):e0322649. doi: 10.1371/journal.pone.0322649 (PMC12047837; doi:10.1371/journal.pone.0322649)
Supplement: Attachment — Submitted filename: Response_to_reviewers_auresp_2.docx [file pone.0322649.s016.docx]

**Reviewer #1:** The authors have addressed major concerns expressed by the reviewers by modifications to the text and title, and including new figures and references, that have been helpful in significantly improving the manuscript. With regards to a more relevant analysis using heterodimers of Zebrafish S100 proteins, the authors have offered a clear explanation for why experimentally measuring the functional properties of possible homo and heterodimeric combinations would be challenging. Their justification for addressing the issue using a pilot computational approach seems reasonable.

**Response:** Thank you for helping us make important, necessary improvements to our manuscript.

**Reviewer #2:** In this revised manuscript, Orlandi and Harms provided new valuable data and experiments that helped address most of the concerns I had. Importantly, they down-tuned their interpretation about the non-existence of a functional ortholog of calprotectin, starting from a modified title, as not all calprotectin-dependent functions were tested in this study and other receptors for a calprotectin-like S100 protein in zebrafish may exist, which they also clearly stated in the results and discussion section. I also appreciate the new section on the modelling of possible homo- and hetero S100 dimers that now more firmly highlight the fact that none of them would be able to mimic the unique hexaHis site from which human calprotectin gets its antimicrobial properties. Although it would be nice to test these hypotheses experimentally, I do fully understand that this cannot be tested at this stage in the current study. I also acknowledge that the EDTA control has been added in the antimicrobial assays, as well as the test with calprotectin for NFkB activation and the inclusion of the SDS-PAGE gel for assessing calprotectin purity levels and homogeneity. These adds-on greatly ameliorated the current manuscript. Overall, I find that the manuscript has been substantially improved. I however have two remaining concerns:

1) The first one is about the analysis of the RNAseq dataset from tail fin amputation in zebrafish and the interpretation of the data made by the authors regarding the wide up-regulation of several zebrafish s100 genes in response to injury. The authors state that was is represented is the transcriptional upregulation for each gene following injury but it is unclear from which precise data these graphs were built. The published RNAseq data contains datasets from 3 distinct time points following injury (1, 2 and 4 dpa) plus datasets pre-injury. How was the analysis done to compare pre- and post-injury transcriptional profiles? Which data were employed for post-injury (only 1 time point or all 3 merged together?). This should be detailed in the experimental section as well as in the figure legend. We did analyze some of these data ourselves and actually could not find any upregulation of some of the mentioned genes following injury, therefore I find it essential that the procedure for comparison pre/post-injury is clarified.

2) I would still be carefully in stating that no functional ortholog of calprotectin for the inflammatory function of this protein are found in zebrafish. I do agree with this conclusion for the antimicrobial activity and for the infection-related pro-inflammatory function. But as previously stated, in mammals, S100A8/A9 exerts its pro-inflammatory activity in many disease contexts and through many different receptors, that can obviously not all be tested in this study. Therefore it should be more strongly emphasized in the discussion/conclusion but also in the abstract. I still don’t think that a strong conclusion can be made on the role pro-inflammatory role of zebrafish s100 proteins and whether might still have a similar function to human calprotectin in that respect.

In conclusion, while the current manuscript has been strongly improved, I would still like to see these two points addressed before it can be considered suitable for publication in PLoS ONE.

**Response:** Thank you for the feedback and for your input which greatly improved our manuscript. To address your concerns, we have made the following revisions:

1) You have made an excellent point. We did not clearly describe how we interpreted the injury dataset and, while doing this revision, we discovered that we had made some incorrect assumptions about the presentation of data on the interactive platform for this dataset. We have now included two more panels in our S3 Fig which parse the data over sample stage: pre-injury, 1-day post-amputation (dpa), 2dpa, and 4dpa. We also amended the language in the results section (lines 252-275) and S3 Figure legend (lines 1080-1091). We now assess gene expression profiles across sample timepoints and compare them to the macrophage marker *mpeg1.1* expression profile. We hope this has resolved your concerns.
2) We understand your worry here since we have only tested a handful of s100 proteins to activate a singular pro-inflammatory pathway. We have made several changes to the text to address this concern, including in the abstract (line 46 and 48-49), introduction (lines 102, 106-109), and results (lines 436-439). We feel that we have sufficiently addressed this point in our discussion (lines 514-519, and 525-556). As you have reiterated, we agree we have not proven that zebrafish s100s did not convergently evolve functions similar to calprotectin. Rather, we find no evidence to support this hypothesis of convergent evolution. But we do stand by our most important claim: that zebrafish do not have an ortholog of calprotectin.

Thank you so much for all of your thoughtful comments and critiques. Our paper has improved significantly during this review process. We greatly appreciate your effort and time.
